# Design, Synthesis, Molecular Modeling Study and Biological Evaluation of New N'-Arylidene-pyrido [2,3-*d*]pyrimidine-5-carbohydrazide Derivatives as Anti-HIV-1 Agents

**DOI:** 10.22037/ijpr.2019.112198.13597

**Published:** 2019

**Authors:** Elnaz Ebrahimzadeh, Seyyed Abbas Tabatabai, Rouhollah Vahabpour, Zahra Hajimahdi, Afshin Zarghi

**Affiliations:** a *Department of Medicinal Chemistry, School of Pharmacy, Shahid Beheshti University of Medical Sciences, Tehran, Iran. *; b *Medical Lab Technology Department, School of Allied Medical Sciences, Shahid Beheshti University of Medical Sciences, Tehran, Iran.*

**Keywords:** Synthesis, Pyridopyrimidine-5-carbohydrazide, HIV-1 integrase, Molecular modeling, Anti-HIV-1

## Abstract

In an attempt to identify potential new agents that are active against HIV-1, a series of novel pyridopyrimidine-5-carbohydrazide derivatives featuring a substituted benzylidene fragment were designed and synthesized based on the general pharmacophore of HIV-1 integrase inhibitors. The cytotoxicity profiles of these compounds showed no significant toxicity to human cells and they exhibited anti-HIV-1 activity with EC50 values ranging from 90 to 155 µM. Compound 5j bearing 4-methylbenzylidene group was found to be the most active compound with EC50 = 90 µM and selectivity index, CC50/EC50 = 6.4. Molecular modeling studies indicated the capacity of compound 5j to interact with two Mg2+ cations and several residues that are important in HIV-1 integrase inhibition. These findings suggested that pyridopyrimidine-5-carbohydrazide scaffold might become a promising template for development of novel anti-HIV-1 agents.

## Introduction

**Figure 1.**
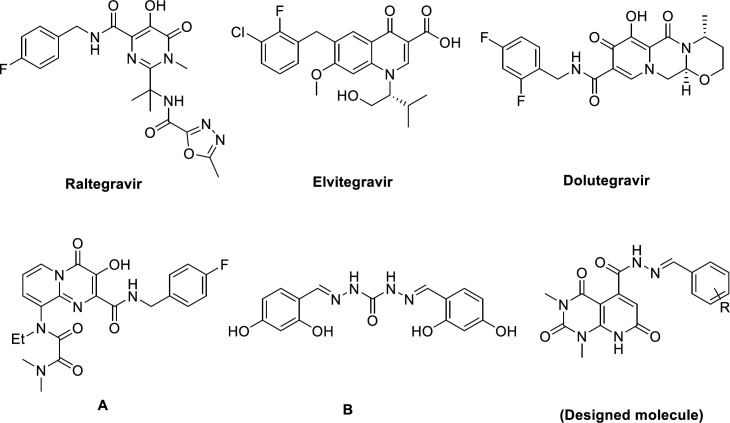
FDA-approved HIV integrase inhibitors (Raltegravir, Elvitegravir, Dolutegravir), HIV IN inhibitors (**A** and **B**) and our designed molecule

Acquired immunodeficiency syndrome (AIDS) is a chronic and life-threatening disease caused by the human immunodeficiency virus (HIV). As reported by World Health Organization (WHO) globally, 37 million people living with HIV and about 0.9 million people died from illnesses incidental to AIDS in 2017 (1). Highly active antiretroviral therapy (HAART), combining drugs that target different stages of HIV life cycle, diminish replication of HIV-1 and lengthens AIDS-free survival, at least temporarily (2, 3). However, factors like rising of multidrug resistant, drug-drug interaction, poor bioavailability, and cumulative toxicities cause a continuous need for alternative strategies and new targets for controlling HIV infection (4, 5). HIV integrase, one of the three fundamental enzymes for HIV replication (besides reverse transcriptase and protease) catalyzes integration of viral DNA in two distinct reactions: 3′ processing (3′P) in which an endonuclelyotic cleavage generates two CA-3′-OH at each end of viral DNA; strand transfer reaction (ST), leads to the insertion of the processed viral DNA into the host DNA (6, 7). Thanks to no host functional analogue and its essential role in viral replication, it is a propitious target for designing novel inhibitors with high selectivity and low toxicity (8, 9). Several efforts have been made to design different structural class of integrase inhibitors (INIs) like diketo acids and their bioisosters (10-13), leading to three FDA approved drugs in this class: Raltegravir, Elvitegravir, and Dolutegravir (Figure 1) (14-17). The functional principle of these drugs is binding to the two Mg^2+^ ions in the active site of the integrase, comprising a triad of invariant acidic residues, referred to as the DDE motif (Asp64, Asp116, and Glu152) and coordinate those metal ions, and concurrently stacks against the 3′-adenosine (A17) of the viral DNA (18, 19).

Pyridopyrimidine engages a remarkable position in medicinal chemistry as it’s a prominent pharmacophore that demonstrate plethora of biological activities e.g. anticancer (20, 21), anti-inflammatory (22), antimalarial (23), antifungal (24), antimicrobial (25), antiviral activity (26, 27), and so on. Pyridopyrimidines featuring carbonyl moiety are noteworthy scaffolds in design and development of integrase inhibitors because of their metal binding properties (28) (compound **A**, Figure 1). Additionally, Hong *et al.* reported carbohydrazide containing compounds as IN inhibitors (29) (compound **B**, Figure 1). Choosing carbohydrazide-type framework for structural optimization could be due to following aspects: i) as a common building block, carbohydrazide can easily achieve molecular diversity by reacting with a variety of chemical substances, such as carboxylic acid, aldehyde, anhydride acyl chloride, and ester under mild conditions; ii) the streochemical nature of carbohydrazide may properly orient and anchor the conjugated hydrophobic subunit into the key binding pocket groove via a favorable and stable configuration (30). Therefore, we selected pyridopyrimidine-5-carbohydrazide as a core structure to design new anti-HIV-1 compounds and attached it to an arylidene fragment to occupy the hydrophobic pocket of IN active site and explore the effect of it on anti-HIV-1 activity. We also performed docking studies to predict the interaction of new synthesized compounds with the active site of integrase and their probable mechanism of action.

## Experimental

*Chemistry*

*General*

All chemicals and solvents used in this study were purchased from Merck AG and Aldrich Chemical. Melting points (mp) were determined with a Thomas–Hoover capillary apparatus. Infrared spectra were acquired using a Perkin Elmer Model 1420 spectrometer. A Bruker FT-400 MHz instrument (Bruker Biosciences, USA) was used to acquire ^1^HNMR spectra with TMS as internal standard. DMSO-*d6* was used as solvent. Coupling constant (*J*) values are estimated in hertz (Hz) and spin multiples are given as s (singlet), d (double), t (triplet), q (quartet), m (multiplet), and br (broad). The mass spectral measurements were performed on a 6410 Agilent LCMS triple quadrupole mass spectrometer (LCMS) with an electrospray ionization (ESI) interface. Microanalyses, determined for C and H, were within ± 0.4% of theoretical values.

*Methyl 1,3-dimethyl-2,4,7-trioxo-1,2,3,4, 7,8-hexahydropyrido[2,3-d]pyrimidine-5-carboxylate (* ***2*** *)*

A solution of 6-amino-1,3-dimethy-luracil **1** (10 mmol), and dimethyl acetylene-dicarboxylate (DMAD) (11 mmol) in methanol (50 mL) was refluxed overnight. The mixture was filtered hot, and the filtrate condensed to a small volume. The residue was filtered and recrystallized from methanol. Yield, 63%; yellow powder; mp: 202.3-203.9°C; IR (KBr disk) v (cm^-1^) 1654-1734 (C=O), 3775 (N-H); LCMS (ESI) *m/z* 288 (M+Na^+^, 100).

*1,3-Dimethyl-2,4,7-trioxo-1,2,3,4,7,8-hexahydropyrido[2,3-d]pyrimidine-5-carbohydrazide (* ***3*** *)*

A mixture of compound **2** (5 mmol) and hydrazine hydrate (about 100% N_2_H_5_OH, for synthesis grade) (50 mmol) in absolute ethanol (50 mL) was refluxed for 24 h. After reaction completion, the mixture was filtered off and washed with ethanol. Yield, 67%; white powder; mp: 231 °C (decomposed); IR (KBr): v (cm^-1^) 1734, 1704, 1658, 1645 (C=O), 3168, 2295 (NH), 3306 (NH_2_); LC-MS (ESI) *m/z:* 266 (M+H^+^, 100).

*General procedure for the synthesis of N’-arylidene-1,3-dimethyl-2,4,7-trioxo-1,2,3,4,7,8-hexahydropyrido[2,3-d]pyrimidine-5-carbohydrazides (* ***5a-l*** *)*

A mixture of compound **3** (1 mmol) and substituted benzaldehydes **4** (1 mmol) in absolute ethanol (20 mL) was refluxed for 12 h. Completion of the reaction was monitored by TLC (Thin Layer Chromatography). The reaction mixture was cooled, filtered off and the product was recrystallized from chloroform and methanol. 

*N’-benzylidene-1,3-dimethyl-2,4,7-trioxo-1,2,3,4,7,8-hexahydropyrido[2,3-d]pyrimidine-5-carbohydrazide (* ***5a*** *)*

Yield, 65%; creamy powder; mp: 286 °C (decomposed); IR (KBr): v(cm^-1^) 1400–1600 (aromatic), 1652 (C=O, ring), 1704 (C=O), 3079, 3228 (NH); ^1^HNMR (DMSO-*d*_6_, 400 MHz); mixture of E and Z isomers: δ ppm 3.19 & 3.24 (s, 3H, N_1_CH_3_), 3.55 (s, 3H, N_3_CH_3_), 6.41 & 6.52 (s, 1H, H_6_), 7.32 (s, 3H, benzylidene H_3_ & H_4_ & H_5_), 7.46 (d, 2H, benzylidene H_2_ & H_6_, *J*=6.2 Hz), 7.73 (d, 2H, benzylidene H_2_ & H_6_, *J*=5.76 Hz), 7.99 & 8.17 (s, 1H, N=CH), 11.67 & 11.94 (s, 1H, N_8_H); LC-MS (ESI) *m/z*: 352 (M-1, 100); Anal. Calcd. for C_17_H_15_N_5_O_4_: C, 57.79; H, 4.28; N, 19.82. Found: C, 57.62; H, 4.09; N, 19.92.

*N’-(2-methoxybenzylidene)-1,3-dimethyl-2,4,7-trioxo-1,2,3,4,7,8-hexahydropyrido[2,3-d]pyrimidine-5-carbohydrazide (* ***5b*** *)*

Yield, 45%; white powder; mp: 231 °C (decomposed); IR (KBr): v (cm^-1^) 1400–1600 (aromatic), 1646 (C=O, ring), 1706 (C=O), 3066, 3227 (NH); ^1^HNMR (DMSO-*d*_6_, 400 MHz); mixture of E and Z isomers: δ ppm 3.19 & 3.24 (s, 3H, N_1_CH_3_), 3.51 & 3.54 (s, 3H, N_3_CH_3_), 3.81 & 3.84 (s, 3H, OCH_3_), 6.39 & 6.51 (s, 1H, H_6_), 6.82 (t, 1H, 2-methoxybenzylidene H_5_, *J=*7.48 Hz), 7.01-7.06 (m, 2H, 2-methoxybenzylidene H_3_ & H_5_), 7.11 (d, 1H, 2-methoxybenzylidene H_3_, *J*=8.36 Hz), 7.16 (d, 1H, 2-methoxybenzylidene H_6_, *J*=7.16 Hz), 7.30 (t, 1H, 2-methoxybenzylidene H_4_, *J*=8.24 Hz), 7.43 (t, 1H, 2-methoxybenzylidene H_4_, *J*=7.44 Hz), 7.87 (d, 1H, 2-methoxybenzylidene H_6_, *J*=7.12 Hz), 8.31 & 8.51 (s, 1H, N=CH), 11.63 & 11.88 (s, 1H, N_8_H); LC-MS (ESI) *m/z*: 382 (M-1, 100); Anal. Calcd. for C_18_H_17_N_5_O_5_: C, 56.39; H, 4.47; N, 18.27. Found: C, 56.55; H, 4.66; N, 18.10.

*N’-(3-methoxybenzylidene)-1,3-dimethyl-2,4,7-trioxo-1,2,3,4,7,8-hexahydropyrido[2,3-d]pyrimidine-5-carbohydrazide (* ***5c*** *)*

Yield, 53%; white powder; mp: 231 °C (decomposed); IR (KBr): v (cm^-1^) 1400–1600 (aromatic), 1652 (C=O, ring), 1704 (C=O), 3079, 3228 (NH); ^1^HNMR (DMSO-*d*_6_, 400 MHz); mixture of E and Z isomers: δ ppm 3.19 & 3.24 (s, 3H, N_1_CH_3_), 3.54 (s, 3H, N_3_CH_3_), 3.64 & 3.82 (s, 3H, OCH_3_), 6.41 & 6.52 (s, 1H, H_6_), 6.81 (s, 1H, 3-methoxybenzylidene H_2_), 6.88-6.92 (m, 2H, 3-methoxybenzylidene H_2_ & H_4_), 7.02 (d, 1H, 3-methoxybenzylidene H_4_, *J*=8.08 Hz), 7.22 (t, 1H, 3-methoxybenzylidene H_5_, *J*=7.92 Hz), 7.29 (d, 1H, 3-methoxybenzylidene H_6_, *J*=8.76 Hz), 7.38 (t, 1H, 3-methoxybenzylidene H_5_, *J*=7.88 Hz), 7.94 & 8.13 (s, 1H, N=CH), 11.71 & 11.97 (s, 1H, N_8_H); LC-MS (ESI) *m/z*: 384 (M+H^+^, 100); Anal. Calcd. for C_18_H_17_N_5_O_5_: C, 56.39; H, 4.47; N, 18.27. Found: C, 56.70; H, 4.69; N, 18.19.

*N’-(4-methoxybenzylidene)-1,3-dimethyl-2,4,7-trioxo-1,2,3,4,7,8-hexahydropyrido[2,3-d]pyrimidine-5-carbohydrazide (* ***5d*** *)*

Yield, 69%; pale yellow powder; mp: 231 °C (decomposed); IR (KBr): v (cm^-1^) 1400–1600 (aromatic), 1662 (C=O, ring), 1703 (C=O), 3076, 3196 (NH); ^1^HNMR (DMSO-*d*_6_): δ ppm 3.19 & 3.24 (s, 3H, N_1_CH_3_), 3.54 & 3.55 (s, 3H, N_3_CH_3_), 3.72 & 3.81 (s, 3H, OCH_3_), 6.39 & 6.50 (s, 1H, H_6_), 6.87 (d, 2H, 4-methoxybenzylidene H_3_ & H_5_, *J*=8.00 Hz), 7.27 (d, 2H, 4-methoxybenzylidene H_2_ & H_6_, *J*=8.00 Hz), 7.67 (d, 2H, 4-methoxybenzylidene H_3_ & H_5_, *J*=8.00 Hz), 7.81 (d, 2H, 4-methoxybenzylidene H_2_ & H_6_, *J*=8.08 Hz), 7.92 & 8.01 (s, 1H, N=CH), 11.54 & 11.82 (s, 1H, N_8_H), 12.38 (brs, 1H, NH); LC-MS (ESI) *m/z*: 382 (M-1, 100); Anal. Calcd. for C_18_H_17_N_5_O_5_: C, 56.39; H, 4.47; N, 18.27. Found: C, 56.71; H, 4.11; N, 17.98.

*N’-(2-chlorobenzylidene)-1,3-dimethyl-2,4,7-trioxo-1,2,3,4,7,8-hexahydropyrido[2,3-d]pyrimidine-5-carbohydrazide (* ***5e*** *)*

Yield, 60%; white powder; mp: 297 °C (decomposed); IR (KBr): v (cm^-1^) 1400–1600 (aromatic), 1657 (C=O, ring), 1701 (C=O), 3085, 3245 (NH); ^1^HNMR (DMSO-*d*_6_, 400 MHz); mixture of E and Z isomers: δ ppm 3.19 & 3.25 (s, 3H, N_1_CH_3_), 3.54 (s, 3H, N_3_CH_3_), 6.43 & 6.54 (s, 1H, H_6_), 7.24-7.53 (m, 4H, 2-chlorobenzylidene), 8.37 & 8.58 (s, 1H, N=CH), 11.91 & 12.13 (s, 1H, N_8_H), 12.45 (brs, 1H, NH); LC-MS (ESI) *m/z*: 386 (M-1, 100); Anal. Calcd. for C_17_H_14_ClN_5_O_4_: C, 52.66; H, 3.64; N, 18.06. Found: C, 52.46; H, 3.34; N, 18.22.

*N’-(3-chlorobenzylidene)-1,3-dimethyl-2,4,7-trioxo-1,2,3,4,7,8-hexahydropyrido[2,3-d]pyrimidine-5-carbohydrazide (* ***5f*** *)*

Yield, 60%; white powder; mp: 302 °C (decomposed); IR (KBr): v (cm^-1^) 1400–1600 (aromatic), 1657 (C=O, ring), 1709 (C=O), 3092, 3235 (NH); ^1^HNMR (DMSO-*d*_6_, 400 MHz); mixture of E and Z isomers: δ ppm 3.19 & 3.24 (s, 3H, N_1_CH_3_), 3.55 (s, 3H, N_3_CH_3_), 6.42 & 6.53 (s, 1H, H_6_), 7.28-7.37 (m, 2H, 3-chlorobenzylidene H_4_ & H_5_), 7.50 (d, 1H, 3-chlorobenzylidene H_6_, *J*=5.04 Hz), 7.70 & 7.80 (s, 1H, 3-chlorobenzylidene H_2_) 7.97 & 8.15 (s, 1H, N=CH), 11.83 & 12.09 (s, 1H, N_8_H), 12.28 (brs, 1H, NH); LC-MS (ESI) *m/z:* 386 (M-1, 100); Anal. Calcd. for C_17_H_14_ClN_5_O_4_: C, 52.66; H, 3.64; N, 18.06. Found:

*N’-(4-chlorobenzylidene)-1,3-dimethyl-2,4,7-trioxo-1,2,3,4,7,8-hexahydropyrido[2,3-d]pyrimidine-5-carbohydrazide (* ***5g*** *)*

Yield, 57%; white powder; mp: 305 °C (decomposed); IR (KBr): v (cm^-1^) 1400–1600 (aromatic), 1641 (C=O, ring), 1719 (C=O), 2929, 3267 (NH); ^1^HNMR (DMSO-*d*_6_, 400 MHz); mixture of E and Z isomers: δ ppm 3.19 & 3.24 (s, 3H, N_1_CH_3_), 3.54 & 3.55 (s, 3H, N_3_CH_3_), 6.41 & 6.52 (s, 1H, H_6_), 7.36 (dd, 2H, 4-chlorobenzylidene), 7.53 (d, 2H, 4-chlorobenzylidene H_3_ & H_5_, *J*=8.36 Hz), 7.76 (d, 2H, 4-chlorobenzylidene H_2_ & H_6_, *J*=8.36 Hz), 7.98 & 8.16 (s, 1H, N=CH), 11.76 & 12.02 (s, 1H, N_8_H), 12.22 (brs, 1H, NH); LC-MS (ESI) *m/z*: 414 (M+H^+^, 100); Anal. Calcd. for C_17_H_14_ClN_5_O_4_: C, 52.66; H, 3.64; N, 18.06. Found: C, 52.91; H, 3.77; N, 18.16.

*1,3-Dimethyl-N’-(2-methylbenzylidene)-2,4,7-trioxo-1,2,3,4,7,8-hexahydropyrido[2,3-d]pyrimidine-5-carbohydrazide (* ***5h*** *)*

Yield, 51%; white powder; mp: 277 °C (decomposed); IR (KBr): v (cm^-1^) 1400–1600 (aromatic), 1657 (C=O, ring), 1691 (C=O), 3067, 3244 (NH); ^1^HNMR (DMSO-*d*_6_, 400 MHz); mixture of E and Z isomers: δ ppm 2.11 & 2.41 (s, 3H, CH_3_), 3.20 & 3.25 (s, 3H, N_1_CH_3_), 3.54 (s, 3H, N_3_CH_3_), 6.41 & 6.52 (s, 1H, H_6_), 7.10-7.33 (m, 4H, 2-methylbenzylidene), 8.19 & 8.44 (s, 1H, N=CH), 11.65 & 11.87 (s, 1H, N_8_H), 12.22 (brs, 1H, NH); LC-MS (ESI) *m/z*: 414 (M+H^+^, 100); Anal. Calcd. for C_18_H_17_N_5_O_4_: C, 58.85; H, 4.66; N, 19.06. Found: C, 59.05; H, 4.84; N, 19.12. 

*1,3-Dimethyl-N’-(3-methylbenzylidene)-2,4,7-trioxo-1,2,3,4,7,8-hexahydropyrido[2,3-d]pyrimidine-5-carbohydrazide (* ***5i*** *)*

Yield, 62%; white powder; mp: 280 °C (decomposed); IR (KBr): v (cm^-1^) 1400–1600 (aromatic), 1661 (C=O, ring), 1713 (C=O), 3096, 3245 (NH); ^1^HNMR (DMSO-*d*_6_, 400 MHz); mixture of E and Z isomers: δ ppm 2.22 & 2.36 (s, 3H, CH_3_), 3.18 & 3.24 (s, 3H, N_1_CH_3_), 3.54 & 3.55 (s, 3H, N_3_CH_3_), 6.41 & 6.51 (s, 1H, H_6_), 7.12-7.27 (m, 4H, 3-methylbenzyl), 7.26 (d, 1H, 3-methylbenzylidene H_4_, *J*=7.52 Hz), 7.35 (t, 1H, 3-methylbenzylidene H_5_, *J*=7.6 Hz), 7.51 (d, 1H, 3-methylbenzylidene H_6_, *J*=7.6 Hz), 7.56 (s, 1H, 3-methylbenzylidene H_2_), 7.95 & 8.12 (s, 1H, N=CH), 11.67 & 11.92 (s, 1H, N_8_H), 12.18 (brs, 1H, NH); LC-MS (ESI) *m/z*: 366 (M-1, 100); Anal. Calcd. for C_18_H_17_N_5_O_4_: C, 58.85; H, 4.66; N, 19.06. Found: 

*1,3-Dimethyl-N’-(4-methylbenzylidene)-2,4,7-trioxo-1,2,3,4,7,8-hexahydropyrido[2,3-d]pyrimidine-5-carbohydrazide (* ***5j*** *)*

Yield, 71%; white powder; mp: 284 °C (decomposed); IR (KBr): v (cm^-1^) 1400–1600 (aromatic), 1646 (C=O, ring), 1718 (C=O), 2944, 3254 (NH); ^1^HNMR (DMSO-*d*_6_, 400 MHz); mixture of E and Z isomers: δ ppm 2.16 & 2.26 (s, 3H, CH_3_), 3.09 & 3.15 (s, 3H, N_1_CH_3_), 3.45 & 3.46 (s, 3H, N_3_CH_3_), 6.31 & 6.42 (s, 1H, H_6_), 7.03 (d, 2H, 4-methylbenzylidene H_3_ & H_5_, *J*=8.00 Hz), 7.12 (d, 2H, 4-methylbenzylidene H_2_ & H_6_, *J*=8.00 Hz), 7.19 (d, 2H, 4-methylbenzylidene H_3_ & H_5_, *J*=8.00 Hz), 7.53 (d, 2H, 4-methylbenzylidene H_2_ & H_6_, *J*=8.08 Hz), 7.86 & 8.04 (s, 1H, N=CH), 11.52 & 11.78 (s, 1H, N_8_H), 12.26 (brs, 1H, NH); LC-MS (ESI) *m/z*: 368 (M+H^+^, 100); Anal. Calcd. for C_18_H_17_N_5_O_4_: C, 58.85; H, 4.66; N, 19.06. Found: C, 58.59; H, 4.31; N, 19.30.

*N’-(3-fluorobenzylidene)-1,3-dimethyl-2,4,7-trioxo-1,2,3,4,7,8-hexahydropyrido[2,3-d]pyrimidine-5-carbohydrazide (* ***5k*** *)*

Yield, 62%; yellow powder; mp: 287 °C (decomposed); IR (KBr): v (cm^-1^) 1400–1600 (aromatic), 1654 (C=O, ring), 1702 (C=O), 3086, 3247 (NH); ^1^HNMR (DMSO-*d*_6_, 400 MHz); mixture of E and Z isomers: δ ppm 3.19 & 3.24 (s, 3H, N_1_CH_3_), 3.54 & 3.55 (s, 3H, N_3_CH_3_), 6.41 & 6.53 (s, 1H, H_6_), 7.09-7.20 (m, 4H, 3-fluorobenzylidene H_4_ & H_5_), 7.50 (d, 1H, 3-fluorobenzylidene H_6_, *J*=5.04 Hz), 7.27-7.39 (m, 1H, 3-fluorobenzylidene H_6_), 7.49-7.60 (m, 1H, 3-fluorobenzylidene H_2_), 7.98 & 8.17 (s, 1H, N=CH), 11.81 & 12.08 (s, 1H, N_8_H); LC-MS (ESI) *m/z*: 370 (M-1, 100); Anal. Calcd. for C_17_H_14_FN_5_O_4_: C, 54.99; H, 3.80; N, 18.86. Found: C, 55.19; H, 3.96; N, 18.59.

*N’-(4-fluorobenzylidene)-1,3-dimethyl-2,4,7-trioxo-1,2,3,4,7,8-hexahydropyrido[2,3-d]pyrimidine-5-carbohydrazide (* ***5l*** *)*

Yield, 52%; creamy powder; mp: 220.7- 221.3 °C; IR (KBr): v (cm^-1^) 1400–1600 (aromatic), 1658 (C=O, ring), 1715 (C=O), 2973, 3087 (NH); ^1^HNMR (DMSO-*d*_6_, 400 MHz); mixture of E and Z isomers: δ ppm 3.19 & 3.24 (s, 3H, N_1_CH_3_), 3.54 & 3.55 (s, 3H, N_3_CH_3_), 6.40 & 6.51 (s, 1H, H_6_), 7.16 (t, 2H, 4-fluorobenzylidene H_3_ & H_5_, *J*=8.76), 7.31 (t, 2H, 4-fluorobenzylidene H_3_ & H_5_, *J*=8.72), 7.37-7.40 (m, 2H, 4-fluorobenzylidene H_2_ & H_6_), 7.78-7.81 (m, 2H, 4-fluorobenzylidene H_2_ & H_6_), 7.98 & 8.16 (s, 1H, N=CH), 11.70 & 12.00 (s, 1H, N_8_H), 12.42 (brs, 1H, NH); LC-MS (ESI) *m/z*: 370 (M-1, 100); Anal. Calcd. for C_17_H_14_FN_5_O_4_: C, 54.99; H, 3.80; N, 18.86. Found: C, 54.78; H, 3.61; N, 18.72.

*Molecular modeling studies*

Molecular modeling was performed using the Autodock Vina (31). Crystal structure of the HIV intasome (pdb code: 3OYA) was used for binding mode analysis of HIV IN inhibitory activity. The protein and ligands were prepared in Autodock tools 1.5.6 from MGL Tools package (32). The co-crystallized ligand and water molecules were extracted, Kollman charges were added, nonpolar hydrogens were merged and AutoDock4 atom type was assigned to the protein structure. The ligand was created and minimized using HyperChem 8.0 (33). The active site was defined as a Grid box around the crystallographic ligand raltegravir in 20×20×20 dimensions. The most active compound was docked in the active site and the bioactive conformations were generated using Autodock Vina.

*In vitro anti-HIV-1 and cytotoxicity assays*

The evaluation of inhibitory effect of synthesized compounds was performed by single-round replication assay which was evaluated in our laboratory and reported previously (34-40). Briefly, Hela cells (6 × 10^3^ per well of 96-wells plate) were incubated with single cycle replicable HIV NL4-3 virions (200 ng p24) in the presence of various concentrations of compounds which were added concurrently with viral infection. The supernatants were collected 72 h postinfection and evaluated for p24 antigen load by capture ELISA (Biomerieux, France). Percentage inhibition of p24 expression in the treated culture was calculated as inhibition rate of p24 (%). The 50% effective concentration (EC_50_) was determined for each compound. XTT proliferation method was performed to evaluate the cellular toxicity of compounds. Toxicities of the compounds against the cells were quantified using XTT (sodium 3-[1 (phenyl aminocarbonyl)-3,4-tetrazolium]-bis(4-methoxy-6-nitro)benzene sulfonic acid) reagent, according to the kit instruction (Roche, Germany) (41, 42). Concentration which reduces proliferation of 50% of cells (CC_50_) was calculated subsequent to determination of p24 load. For EC_50_ and CC_50_ determinations, the compounds were tested at six concentrations (10, 50, 100, 150, 200 and 250 mM) in triplicates.

## Results and Discussion

*Chemistry*

**Figure 2:**
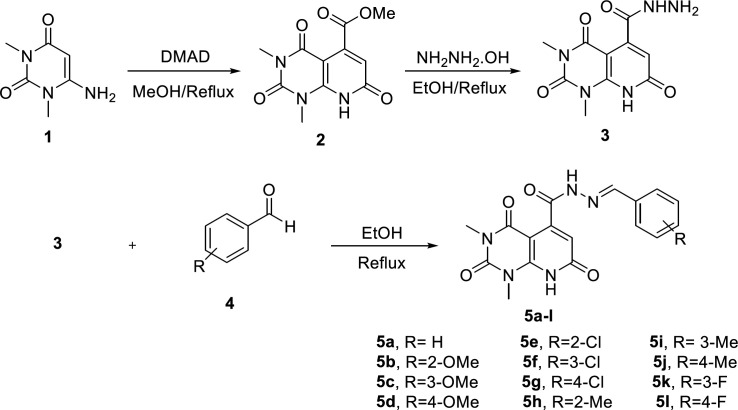
Route of synthesis

The overall synthetic route of target compounds is depicted in Figure 2. The preparation of N’-arylidene-1,3-dimethyl-2,4,7-trioxo-1,2,3,4,7,8-hexahydropyridopyrimidine-5-carbohydrazide analogues (**5a-l**) started with synthesis of ester **2** from the reaction of 6-amino-1,3-dimethyl uracil (**1**) and dimethyl acetylenedicarboxylate (DMAD) in refluxing methanol. Treating **2** with hydrazine hydrate afforded compound **3**. The final compounds **5a-l** were obtained from the reaction of carbohydrazide **3** and substituted aldehydes (**4**) in ethanol. The structure of the synthesized compounds was confirmed by IR, ^1^H-NMR, and ESI-MS.

*Anti-HIV-1 activity evaluation*

All new synthesized compounds were evaluated for their activity against single cycle replicable HIV NL4-3 by determining their ability to inhibit p24 expression in Hela cell cultures. The well-known nucleoside RT inhibitor (AZT) was assayed in the same cells for comparative purpose. They all were then subjected to cell based XTT colorimetric assay for cytotoxicity to ensure that the anti-HIV-1 activity of the compounds was not a result of their cytotoxic effects (43, 44). The biological results are expressed as EC_50_, CC_50_, and SI (selectivity index, given by the CC_50_/EC_50_ ratio). The results are presented in Table 1. As can be seen in Table 1, most of the compounds showed negligible cytotoxicity with CC_50_ > 200 mM. When comparing the anti-HIV-1 activity of tested compounds, it appeared that the presence of substituents on benzylidene fragment confers better inhibition than the unsubstituted counterparts (compound **5a**, EC_50 _>200 mM). In compounds bearing methoxy group as substituent, *meta *and *para* analogues (**5c** and **5d**, respectively) exhibited SI~1 that reflected their cytotoxicity, whereby *ortho* analogue (**5b**) with EC_50_ = 120 mM and SI> 3.75 showed better inhibition. Compound **5k** bearing fluoro substituent showed moderate activity with EC_50_=140 mM and SI= 2.7. Compounds with chloro or methyl substituents were found to be more active than the analogues containing methoxy and fluoro groups. In case of compounds possessing chloro substituent, *para* analogue (**5g**) displayed better anti-HIV activity and selectivity (EC_50_= 110 mM, SI = 4.5) in comparison with *ortho* (compound **5e**, EC_50_= 155 mM) and *meta* (compound **5F**, EC_50_= 120 mM) analogues. Among all tested compounds, the best activity and selectivity was observed with compound **5j** having methyl group at *para*-position (EC_50_= 90 mM, SI = 6.4). The observed structure-activity relationship (SAR) profile revealed that presence of lipophilic substituents such as chloro or particularly methyl conferred an increase in potency and selectivity of the compounds that might be due to good cell penetration in cell-based assays. The best position for introduction of lipophilic substituents was suggested to be *para*-position of phenyl ring maybe because of better interaction with the target. These results indicated that this chemotype could represent a promising scaffold for identifying new anti-HIV agents.

*Molecular modeling studies*

**Figure 3:**
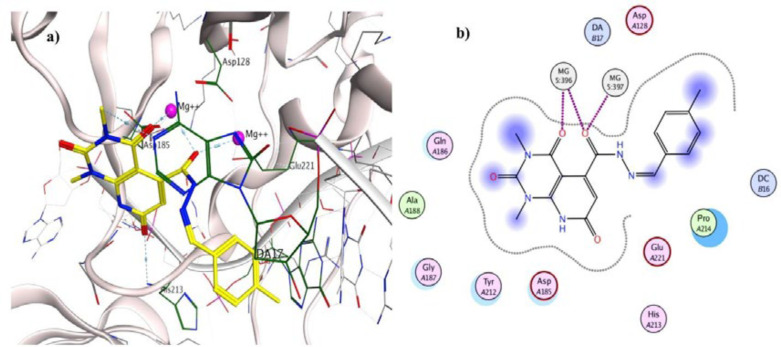
a) 3D alignment of best docked conformer of compound **5j** (shown in yellow) and b) 2D alignment of best docked conformer of compound **5j** in the PFV IN active site

**Figure 4:**
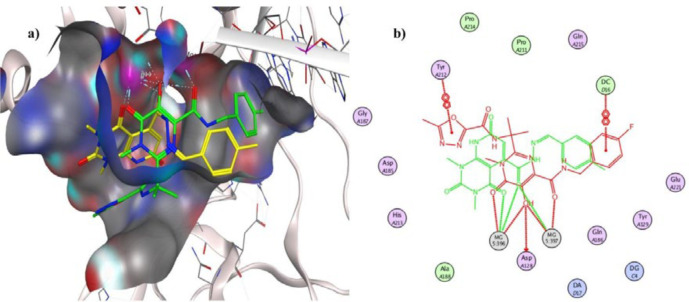
a) Interaction of compound **5j** (shown in yellow) and Raltegravir (shown in green) with the surface of PFV IN active site, b) 2D Superimposition of compound **5j** (shown in green) on Raltegravir (shown in red) in the PFV IN active site

To investigate the binding interactions between synthesized compounds and HIV-1 IN active site, docking studies were carried out. Due to the lack of an experimental X-ray structure of HIV-1 IN/DNA, our studies relied on the crystal structure of prototype foamy virus (PFV) integrase intasome (PDB code: 3OYA) in complex with DNA, 2 Mg^2+^, and Raltegravir at 2.65 Å resolution which is an accepted model for the development of HIV-1 IN inhibitors (19, 45-47). The molecular docking study was performed with Autodock vina, and MOE (Molecular Operating Environment) program was used for visualization and analysis. The 3D and 2D alignment of the most potent compound **5j **in the active site was shown in Figure 3. The predicted binding mode of compound **5j** within the active site suggested potential chelating interactions between oxygen atoms of carbonyl groups and the two Mg^2+^ cofactors with distances of 2.03, 1.84, 2.26, 2.03 Å. Besides, pyridopyrimidine ring involved in a π–stacking interaction with viral base DA17. In addition, 4-methylbenzylidene group occupied the hydrophobic pocket formed by cytosine 16 (C16), guanine 4 (G4) of viral DNA. Potential hydrogen-bonding interaction was also evident between the carbonyl group on position 2 of pyridopyrimidine ring and His213 might help the stability of ligand-enzyme complex. Figure 4 illustrated 2D and 3D superposition of the compound **5j** and the co-crystalized Raltegravir and revealed how well the compound **5j** mimics the binding mode of Raltegravir. These predicted binding modes conform to the general pharmacophore of HIV-1 IN inhibitors; hence the anti-HIV-1 activity of tested compounds might be via HIV-1 IN inhibition. 

**Table 1:** Bioassay data for the compounds **5a-l**, EC_50_ values for inhibition of HIV-1 IN activity, CC_50_ values for toxicity and SI for selectivity index

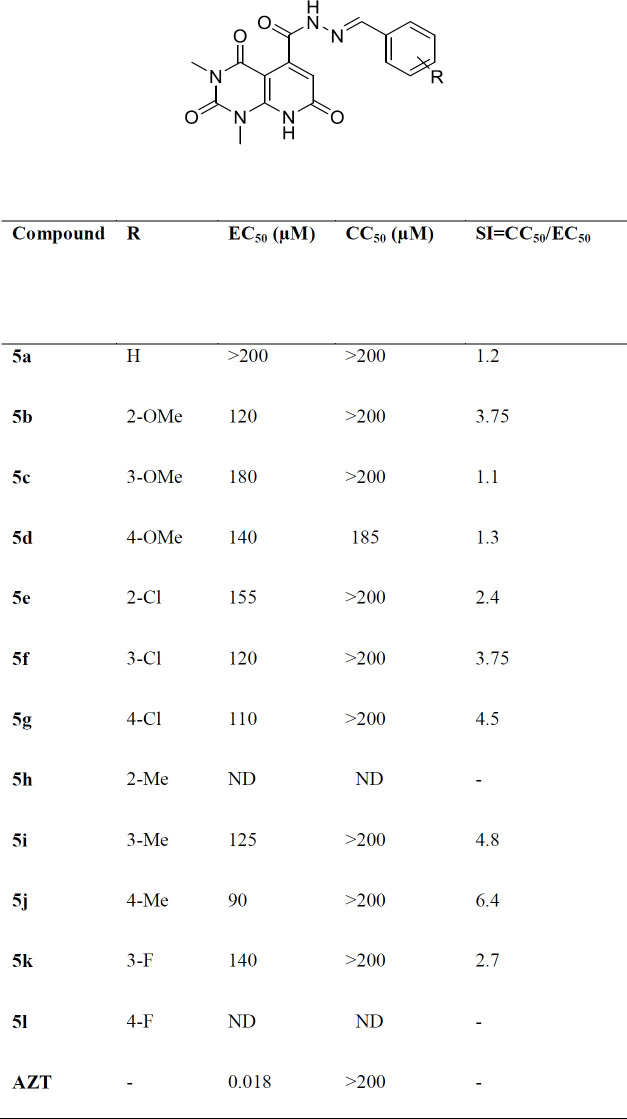

## Conclusion

New pyridopyrimidine-5-carbohydrazides featuring a substituted benzylidene fragment were designed and synthesized on the basis of general pharmacophore of HIV IN inhibitors. In anti-HIV-1 assay, most of the compounds exhibited moderate to good inhibitory activity with EC_50_ values ranging from 90 to 155 mM and no significant cytotoxicity (CC_50_ > 380 mM). The best antiviral activity and selectivity (EC_50_ = 90 mM, SI = 6.4) was observed with compound **5j** bearing 4-methylbenzylidene fragment. The molecular modeling study of compound **5j** predicted a binding model consistent with HIV IN inhibitors. The overall inhibitory and safety profiles of the target compounds suggested that pyridopyrimidine-5-carbohydrazide could serve as a promising scaffold in the development of novel anti-HIV agents. Further structural modifications are needed to optimize potency and selectivity of the compounds. 
